# Natural Blood Feeding and Temperature Shift Modulate the Global Transcriptional Profile of *Rickettsia rickettsii* Infecting Its Tick Vector

**DOI:** 10.1371/journal.pone.0077388

**Published:** 2013-10-14

**Authors:** Maria Fernanda B. M. Galletti, André Fujita, Milton Y. Nishiyama Jr, Camila D. Malossi, Adriano Pinter, João F. Soares, Sirlei Daffre, Marcelo B. Labruna, Andréa C. Fogaça

**Affiliations:** 1 Departamento de Parasitologia, Instituto de Ciências Biomédicas, Universidade de São Paulo, São Paulo, Brazil; 2 Departamento de Ciências da Computação, Instituto de Matemática e Estatística, Universidade de São Paulo, São Paulo, Brazil; 3 Departamento de Bioquímica, Instituto de Química, Universidade de São Paulo, São Paulo, Brazil; 4 Superintendência de Controle de Endemias (SUCEN), São Paulo, Brazil; 5 Departamento de Medicina Veterinária Preventiva e Saúde Animal, Faculdade de Medicina Veterinária e Zootecnia, Universidade de São Paulo, São Paulo, Brazil; University of Minnesota, United States of America

## Abstract

*Rickettsia rickettsii* is an obligate intracellular tick-borne bacterium that causes Rocky Mountain Spotted Fever (RMSF), the most lethal spotted fever rickettsiosis. When an infected starving tick begins blood feeding from a vertebrate host, *R. rickettsii* is exposed to a temperature elevation and to components in the blood meal. These two environmental stimuli have been previously associated with the reactivation of rickettsial virulence in ticks, but the factors responsible for this phenotype conversion have not been completely elucidated. Using customized oligonucleotide microarrays and high-throughput microfluidic qRT-PCR, we analyzed the effects of a 10°C temperature elevation and of a blood meal on the transcriptional profile of *R. rickettsii* infecting the tick *Amblyomma aureolatum*. This is the first study of the transcriptome of a bacterium in the genus *Rickettsia* infecting a natural tick vector. Although both stimuli significantly increased bacterial load, blood feeding had a greater effect, modulating five-fold more genes than the temperature upshift. Certain components of the Type IV Secretion System (T4SS) were up-regulated by blood feeding. This suggests that this important bacterial transport system may be utilized to secrete effectors during the tick vector’s blood meal. Blood feeding also up-regulated the expression of antioxidant enzymes, which might correspond to an attempt by *R. rickettsii* to protect itself against the deleterious effects of free radicals produced by fed ticks. The modulated genes identified in this study, including those encoding hypothetical proteins, require further functional analysis and may have potential as future targets for vaccine development.

## Introduction

The obligate Gram-negative intracellular bacterium *Rickettsia rickettsii* (Order: Rickettsiales, Family: Rickettsiacea) is the etiological agent of Rocky Mountain spotted fever (RMSF), the most lethal spotted fever rickettsiosis. RMSF has been reported in different countries on the American continent (for review, see [1,2]). In Brazil, where the disease is known as Brazilian spotted fever (BSF), a high fatality rate (around 40%) has been recorded [2]. The endemic areas of BSF coincide with the presence of *Amblyomma cajennense* ticks, the major vector for *R. rickettsii*, and of *Amblyomma aureolatum* ticks, an important vector in the metropolitan area of the city of São Paulo [2]. Importantly, in this area, domestic dogs are the primary hosts that sustain natural populations of adult *A. aureolatum* ticks [2].

When an infected starving tick on the ground attaches to the warm skin of a vertebrate host and begins blood feeding, *R. rickettsii* is exposed to a temperature elevation as well as to the components of the blood meal, which is a rich source of nutrients. These factors have been previously associated with the reactivation of rickettsial virulence in ticks [3–6]. However, the mechanisms responsible for this phenotype conversion have not been completely elucidated. Bacteria significantly alter their transcriptome in response to environmental changes to adapt to different conditions. Therefore, studying the global transcriptional profile of pathogens under specific biological conditions can reveal the role of gene sets or pathways in adaptive processes (for review, see [7,8]).

The effects of temperature shifts and/or nutrient restriction on the gene expression profile of bacteria from the genus *Rickettsia* have been previously reported [9–12]. Nonetheless, all the previous studies were performed using bacteria infecting *in vitro* cultures of vertebrate and/or invertebrate cells. In the current study, we present the first transcriptional profile of *R. rickettsii* infecting a natural vector, the tick *Amblyomma aureolatum*. Using oligonucleotide microarrays and high-throughput microfluidic qPCR, the effects of two environmental stimuli were analyzed: a temperature shift and the acquisition of a blood meal by the tick. Although these two stimuli significantly promoted bacterial replication, blood feeding had a greater effect, also modulating five-fold more genes than the temperature shift. Blood feeding induced the expression of antioxidant enzymes, which might correspond to an attempt by *R. rickettsii* to protect itself against free radicals produced by fed ticks. Genes encoding Type IV Secretion System (T4SS) components were also up-regulated by blood feeding, suggesting that this system might play a role in secreting rickettsial effectors during the acquisition of a blood meal by a tick vector. Interestingly, RT-qPCR data showed that the effects of blood feeding combined to temperature upshift is similar to the effects of blood feeding alone, strengthening that the acquisition of the blood meal by the tick triggers major alterations on *R. rickettsii* gene expression. The products of the modulated genes identified in this work, including the hypothetical proteins, should be functionally characterized and may have potential as targets for vaccine development.

## Methods

### Ethics statement

The protocol used in this work was approved by both Animal Experimentation Ethics Committees of the Faculty of Veterinary Medicine and Zootecnics (University of São Paulo, São Paulo, Brazil) and the Institute of Biomedical Sciences (University of São Paulo, São Paulo, Brazil). Guinea pigs and rabbits were purchased from commercial breeders that produce these animals for research use only. Guinea pigs were bought from Anilab (Paulínea, São Paulo, Brazil) and rabbits were bought from Criex (Mogi das Cruzes, São Paulo, Brazil). Domestic dogs were obtained from the Department of Preventive Veterinary Medicine and Animal Health of the University of São Paulo under the coordination of one of the co-authors (MBL) of the present study. These dogs are bred for research use only. Animals are euthanized through the intraperitoneal injection of a combination of ketamine and xylazine.

### Rickettsia rickettsii and Amblyomma aureolatum

In this study, we used the virulent Taiaçu strain of *R. rickettsii* that was originally isolated from a naturally infected *A. aureolatum* tick by inoculating guinea pigs with the tick homogenate [13]. Since its isolation, this strain has been maintained via cryopreservation of infected organs from the guinea pigs.

We also used adult females from the fifth generation of an *A. aureolatum* laboratory colony, which started with engorged females (F_0_) collected from domestic dogs in Atibaia in the state of São Paulo. Ticks from this colony were first infected with the Taiaçu strain of *R. rickettsii* by feeding F_1_ larvae on guinea pigs inoculated with the cryopreserved organs from the infected guinea pigs, as detailed in [14]. Therefore, ticks were exposed to a *R. rickettsii* strain that has never been cultured *in vitro*. During the previous four laboratory generations, *R. rickettsii* was maintained by transstadial perpetuation and transovarial transmission in 100% of the individuals in this colony [15]. In addition, *R. rickettsii* was shown to be highly virulent; all the guinea pigs and rabbits that served as hosts during the larval, nymph, or adult stages in the F_1_ through F_4_ generations developed severe clinical illness, with a 50% fatality rate among the guinea pigs [15]. Off-host developmental stages were held in an incubator at 25°C and 95% relative humidity (RH).

### Exposure to environmental stimuli

*R. rickettsii*-infected unfed adult females (F_5_) were incubated at either 25°C (Group 1, G1) or 35°C (Group 2, G2) for 3 days or fed on domestic dogs (*Canis familiaris*) for 3 days (Group 3, G3). Canine infestations were performed as previously described [15] except the ticks were manually detached from the dogs on the third feeding day. Immediately after incubation or feeding, the ticks were washed in 70% ethanol and sterile phosphate-buffered saline (PBS) (10 mM NaH_2_PO_4_, 1.8 mM KH_2_PO_4_, 140 mM NaCl, and 2.7 mM KCl, pH 7.4) for 10 minutes each. To collect organs, the tick cuticle was carefully cut, and the integument was separated from the internal organs, which were transferred to 100 µL RNAlater^*®*^ Solution (Life Technologies, USA).

### DNA and RNA extraction

Genomic DNA (gDNA) and total RNA from the organs of each individual tick were obtained simultaneously using the InviTrap^®^ TwinSpin Cell Mini Kit (STRATEC, Germany) according to the manufacturer’s instructions.

### Real-time quantitative PCR (qPCR) to quantify *R. rickettsii*

The total number of rickettsiae per adult female tick was quantified by real-time qPCR using specific primers and a TaqMan^®^ probe for the citrate synthase gene (*gltA*) as previously described [16] except for a few modifications, including deleting two bases in the sense primer [17]. Briefly, 8 pmol of each *gltA*-specific primer (sense and antisense), 0.2 pmol of probe and 100 ng of gDNA were added to 10 µL of Quantimix Easy Probes Kit (Biotools, Spain), and the reaction volume was brought to 20 µL with ultrapure water. The reactions were performed on a Mastercycler® ep Realplex^2^ detection system (Eppendorf, Germany) using the following thermocycler program: 2 minutes at 95°C followed by 40 cycles of 15 seconds at 95°C, 15 seconds at 55°C, and 20 seconds at 68°C. Three technical replicates were analyzed for each sample.

### Microarray design

The complete genome sequence of the Sheila Smith strain of *R. rickettsii* (GenBank CP000848.1) [18] was submitted to the Agilent eArray web tool for gene expression probe design (https://earray.chem.agilent.com/earray/). Subsequently, a total of 13,049 60-mer probes representing 99.5% of the bacterial genome (at least six probes per coding sequence) were spotted on an 8 x 15k chip.

### RNA amplification and labeling

Each of the two biological replicates used in the microarray experiments contained total RNA from seven adult females harboring between 1.6 x 10^8^ and 2.2 x 10^8^ bacteria. After DNaseI (Promega, USA) treatment, RNA was reverse transcribed and amplified using the MessageAmp™ II – Bacteria RNA Amplification Kit (Life Technologies, USA) according to the manufacturer’s instructions. To obtain technical replicates (dye swap), the resulting amplified RNA (aRNA) was divided into two samples that were independently labeled with Cy3 or Cy5 using the Genomic DNA ULS^TM^ Labeling Kit (Agilent Technologies, USA). After labeling, G2 samples were mixed with either G1 (to evaluate the effects of a temperature shift) or G3 (to evaluate the effects of blood feeding) samples and hybridized to the microarrays according to the Gene Expression FFPE protocol (Agilent Technologies, USA). Total RNA obtained from non-infected ticks was also similarly processed and hybridized to the *R. rickettsii* microarray to check probe specificity. The coding sequences (CDS) represented by microarray oligonucleotides that cross-hybridized with non-infected *A. aureolatum* transcripts were removed from the data analysis.

### Microarray scanning and data analyses

The chips were scanned on a High-Resolution C Scanner (Agilent Technologies, USA). Images were visually inspected in order to detect eventual air bubbles and dust. Both signal data extraction and spot quality control were carried out using Feature Extraction 10.5 (Agilent Technologies) with default parameters. Spots with low quality, identified by Feature Extraction 10.5, were set to “not available” (NA) and were removed from the dataset. Data were normalized using the loess algorithm as implemented previously [19]. Fold-changes were obtained by calculating the mean value of a gene (summation of normalized probe intensity divided by the number of probes for each gene) and subsequently by calculating the ratios of the normalized intensities of the G2/G1 and G3/G2 samples. Only genes where 65% of the probes exhibited the same transcriptional pattern and a fold-change ≥ 1.5 in both biological replicates were considered to be modulated.

A detailed microarray description can be found in the NCBI Gene Expression Omnibus (GEO) database under Accession Number GPL16681. The complete dataset (Accession Number GSE44349) is publicly available according to MIAME guidelines [19].

### Conserved domain analysis

The complete list of the modulated hypothetical proteins was screened for the presence of conserved domains using The NCBI Conserved Domain Database (CDD, http://www.ncbi.nlm.nih.gov/cdd).

### Microfluidic high-throughput RT-qPCR

To validate the microarray data, specific primers for 85 selected genes (Table S1) were designed using Primer3 [20] and synthesized by Life Technologies (USA). Two hundred ng of the RNA extracted from total organs of one *A. aureolatum* were used as template for reverse transcription (RT) in complementary DNA (cDNA) using M-MLV Reverse Transcriptase (Life Technologies, USA) according to manufacturer’s protocol. Twenty biological replicates (adult females harboring between 2.0 x 10^8^ and 5.7 x 10^8^ rickettisiae) from each biological group (G1, G2 and G3) were analyzed. cDNA was pre-amplified using a mixture containing the 85 primer pairs (200nM each) and the TaqMan^®^ PreAmp Master Mix 2x (Life Technologies, USA). Amplification reactions were carried out on a Mastercycler^®^ Gradient (Eppendorf, Germany) with a thermocycler program of 95°C for 10 minutes and 14 cycles of 95°C for 15 seconds and 60°C for 4 minutes each.  Pre-amplified cDNA samples and primers were combined in wells of a 96x96 chip (Fluidigm, USA) using the integrated fluidic circuit (IFC) controller (Fluidigm, USA) following manufacturer’s instructions. Reactions were performed on a BioMark™ reader (Fluidigm, USA) using a thermocycler program of 50°C for 2 minutes, 70°C for 30 minutes, 25°C for 10 minutes, 50°C for 120 minutes and 95°C for 10 minutes, followed by 35 cycles of 95°C for 15 seconds and 60°C for 60 seconds. In order to exclude non-specific primers, the same procedure was performed using pre-amplified cDNA samples of non-infected females. To determine the efficiency of each primer pair, standard curves were generated using five serial dilutions (1:1, 1:2, 1:4, 1:8, 1:16) of a unique cDNA sample composed by a pool of pre-amplified cDNAs from all biological samples. Only primers presenting efficiencies between 90-100% were considered.

The threshold cycle (Ct) was determined using the BioMark™ qPCR analysis software (Fluidigm, USA). Threshold values were normalized according to the Ct of the reference gene (methionyl-tRNA synthetase), which is expressed at similar levels under all conditions tested, according to our microarray and RT-qPCR data. The 2^-∆∆Ct^ equation [20] was used to calculate the relative expression of select genes in G2 versus G1 (to evaluate the effects of a temperature shift), G3 versus G2 (to evaluate the effects of exposure to blood feeding), and G3 versus G1 (to evaluate the combined effects of both stimuli). To identify the outliers, we considered the Tukey criterion [22]. The medians of fold-changes of samples from the same biological group were analyzed using the Wilcoxon test to statistically validate the differentially expressed genes. *P*-values were corrected for multiple comparisons by the False Discovery Rate (FDR) method [21]. Data was considered significantly different at *p*<0.05.

## Results

### Global gene expression analysis

**Figure 1:**
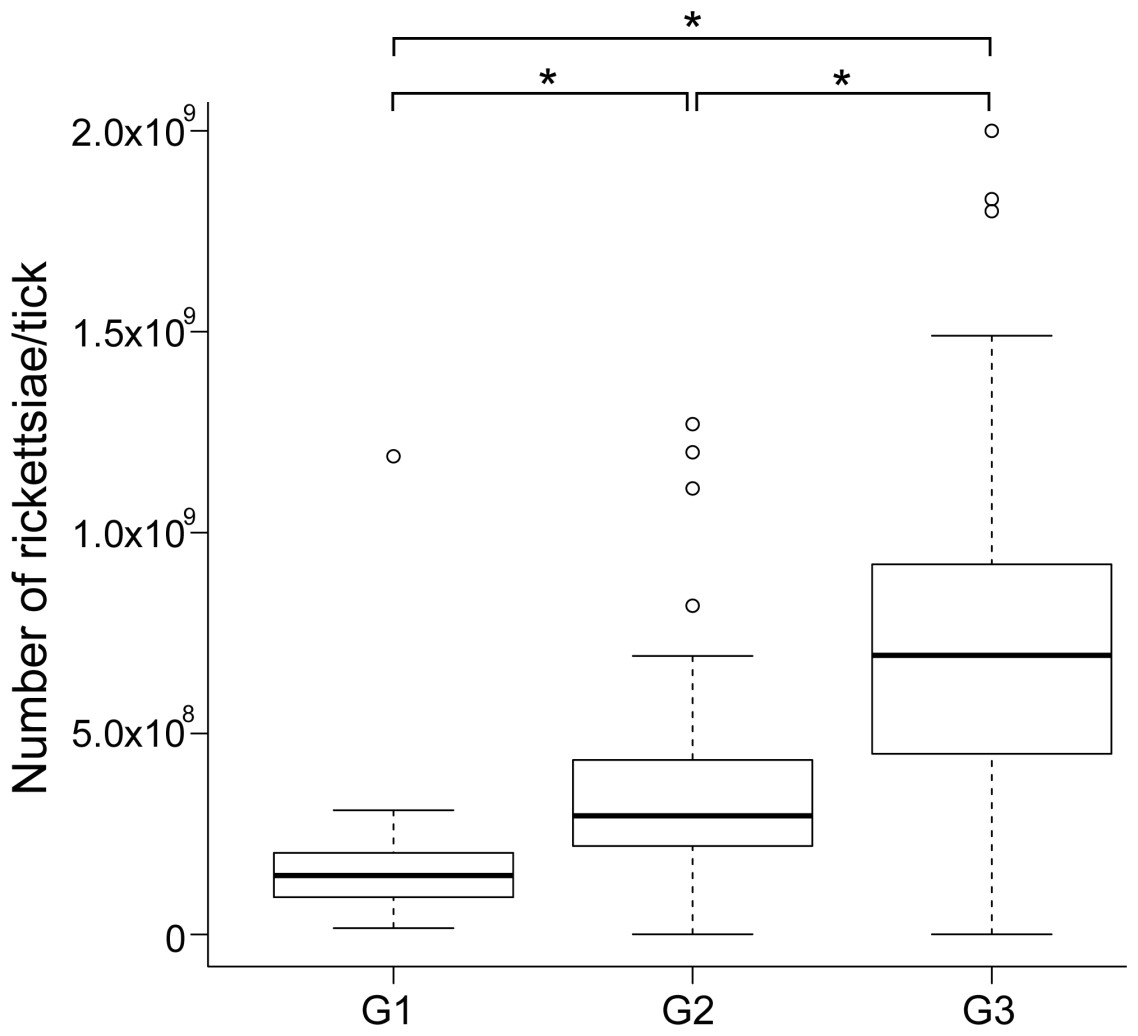
Boxplot representation of *R. rickettsii* load in *A. aureolatum* adult females. The bacterial load in unfed ticks incubated at either 25°C (G1) or 35°C (G2) or fed on dogs (G3) was determined by qPCR using specific primers and a TaqMan probe for *gltA*. The medians are represented by thicker black lines. Outliers are represented by empty circles. *p<0.001, Wilcoxon test.

To obtain *Rickettsia rickettsii* samples for the transcriptional studies, we performed a laboratory-controlled blood feeding infection of ticks with the virulent Taiaçu strain. *Amblyomma aureolatum* F_1_ larvae were fed on experimentally infected *Cavia porcellus* and reared through five consecutive generations in the laboratory. After the F_5_ adults emerged, unfed females were incubated at either 25°C (G1) or 35°C (G2) for 3 days or were fed on dogs (G3) for 3 days. Real-time quantitative PCR demonstrated that 100% of the ticks in the three biological groups were infected with *R. rickettsii*. Moreover, the bacterial load in G3 (7.2 ± 4.0 x 10^8^) and in G2 (3.4 ± 2.2 x 10^8^) is higher than in G1 (2.2 ± 2.0 x 10^8^) ticks (Figure 1), suggesting that both temperature upshift and blood feeding stimulated rickettsial replication.

Total RNA from infected ticks was reverse transcribed, amplified, and labeled for hybridization to oligonucleotide microarrays representing 99.5% of the 1,345 CDS of the Sheila Smith strain of *R. rickettsii*. As a control, total RNA extracted from uninfected ticks was similarly processed and hybridized to *R. rickettsii* microarrays. CDS represented by probes that cross-hybridized with the control transcripts (5.7%) were removed from the data analysis (data not shown). A similar percentage of cross-hybridization with host-cell transcripts was observed by [12].

Microarray screening showed that a 10°C temperature increase modulated the expression of 44 rickettsial genes with a fold-change ≥ 1.5, of which 14 encoded proteins with annotated function (Table S2) and 30 encoded hypothetical proteins (Table S3). On the other hand, the acquisition of a blood meal by the tick vector affected the expression of 221 genes, which comprised 118 proteins with annotated function (Table S4) and 103 hypothetical proteins (Table S5). These results indicated that blood feeding substantially affected the *R. rickettsii* gene expression profile, leading to the differential expression of five-fold more genes than the temperature shift.

**Figure 2:**
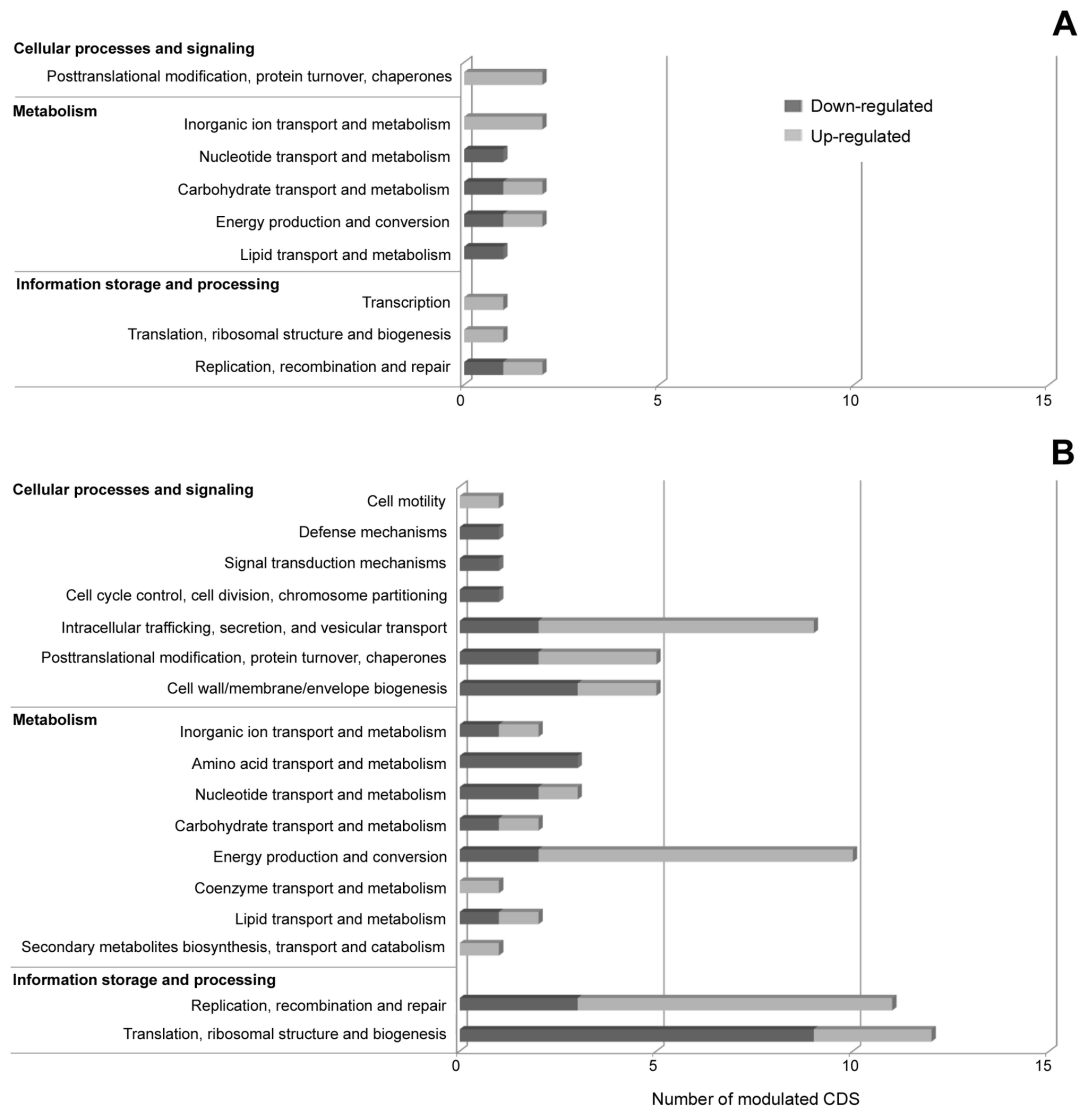
Functional classiﬁcation of *R. rickettsii* genes modulated by exposure to a 10°C increase in incubation temperature (A) or to blood feeding (B).

Among the 44 genes modulated by the temperature increase, 25 were induced and 19 were repressed. The COG (Clusters of Orthologous Groups) functional categories that comprised the majority of the up-regulated genes with annotated function were (i) posttranslational modification and protein turnover and (ii) inorganic ion transport and metabolism (Figure 2A). The down-regulated genes were equally distributed in the following categories: (i) nucleotide, (ii) carbohydrate, and (iii) lipid transport and metabolism; (iv) energy production and conversion; and (ii) replication, recombination, and repair (Figure 2A).

Of the 221 genes differentially expressed in fed compared with unfed ticks in G2, 80 were up-regulated and 141 were down-regulated. Most of the annotated down-regulated genes were involved in translation, ribosomal structure, and biogenesis, whereas the majority of the up-regulated genes were associated with the following categories: (i) energy production and conversion; (ii) replication, recombination, and repair; and (iii) intracellular trafficking, secretion, and vesicular transport (Figure 2B).

Certain up-regulated genes in the last category encode components of the Type IV Secretion System (T4SS; VirB8, VirB9, VirB10, VirB11, and VirD4) (Table S4). Differently, v*irB3*, which encodes another T4SS component, was repressed. Remarkably, three genes encoding the cell surface antigen-like protein Sca8 and one encoding Sca10 were significantly down-regulated (fold-change ≥ 2.0) by blood feeding, and the gene expression of enzymes related to protection against oxidative stress (ferredoxin, glutaredoxin 3, and thioredoxin peroxidase 1) was induced (Table S4).

### *In silico* domain analysis of the hypothetical proteins

As expected, numerous genes encoding hypothetical proteins in *R. rickettsii* were modulated by both temperature change and blood feeding (Tables S3 and S5, respectively). The nucleotide sequences of all the genes encoding hypothetical proteins were submitted for a bioinformatics search against The NCBI Conserved Domain Database (CDD). Of the 133 analyzed sequences, 89 contained domains related to other proteins in the CDD (Tables S3 and S5). Analyzing the hypothetical proteins modulated by temperature change revealed that they contained domains similar to SpoT (A1G_06100) and iron permease (A1G_03065) (Table S3). Interestingly, two hypothetical proteins similar to VirB6 (A1G_00820 and A1G_00825), one to VirB2 (A1G_01380), two to ankyrin repeat proteins (A1G_02840 and A1G_04305), and one to thioredoxin (A1G_00185) were among the hypothetical proteins modulated by blood feeding (Table S5).

### Microarray data validation

In order to validate the microarray data, 35 modulated genes were analyzed by high-throughput microfluidic RT-qPCR (Table 1). In addition, the gene expression of 26 genes that were previously reported to play an important role in bacterial physiology, stress response, and/or virulence [22–33] was also analyzed (Table 2). All the 61 primer pairs presented efficiencies between 90% and 100% and were used to calculate the relative expression in G2 versus G1 (to evaluate the effects of temperature upshift), G3 versus G2 (to evaluate the effects of exposure to blood feeding) and G3 versus G1 (to evaluate the combined effects of both stimuli). No amplification was detected when cDNA from non-infected females was used as template in reactions, attesting primer specificity. There was an overall concordance of 100% between the microarray and the RT-qPCR analyses (Table 1). Interestingly, a similar modulation profile was observed upon blood feeding alone or combined to the temperature upshift for 48 from the 61 analyzed genes (Tables 1 and 2). The other 13 genes presented different patterns or transcription levels were not statistically significant. 

**Table 1:** Transcript levels of selected genes of *R. rickettsii* analyzed by microarrays and high-throughput microfluidic RT-qPCR.

		**Temperature shift**			**Blood feeding**			**Temperature shift + Blood feeding**	
		**Microarray**	**RT-qPCR**		**Microarray**	**RT-qPCR**		**RT-qPCR**	
**Gene ID**	**Annotation**	**Fold change (replicate 1; replicate 2)**	**Fold change**	***p*-value**	**Fold change (replicate 1; replicate 2)**	**Fold change**	***p*-value**	**Fold change**	***p*-value**
A1G_00295	cell surface antigen-like protein Sca10	ND	-1.67	0.0000	**-2.33 (-2.39; -2.27**)	**-4.76**	**0.0000**	-8.33	0.0000
A1G_00515	ADP,ATP carrier protein	ND	NS		**-1.96 (-1.91; -2.02**)	**-2.17**	**0.0000**	-2.13	0.0000
A1G_00520	glycerol-3-phosphate transporter	ND	NS		**-1.73 (-1.62; -1.83**)	**-2.70**	**0.0000**	-2.22	0.0000
A1G_00680	30S ribosomal protein S2	ND	1.41	0.0018	**-2.46 (-2.62; -2.29**)	**-4.00**	**0.0000**	-2.86	0.0000
A1G_00685	elongation factor Ts	ND	NS		**-1.79 (-1.79; -1.78**)	**-2.94**	**0.0000**	-2.70	0.0000
A1G_00810	type IV secretion system protein VirB3	ND	1.45	0.0016	**-1.98 (-2.07; -1.89**)	**-2.78**	**0.0000**	-1.92	0.0000
A1G_01445	cell surface antigen-like protein Sca8	ND	1.43	0.0080	**-5.41 (-5.66; -5.15**)	**-12.5**	**0.0000**	-8.33	0.0000
A1G_01495	ferredoxin	ND	NS		**2.19 (2.30; 2.09**)	**1.36**	**0.0000**	1.29	0.0012
A1G_01525	glutaredoxin 3	ND	-1,25	0.0019	**2.68 (3.12; 2.23**)	**1.93**	**0.0000**	1.54	0.0000
A1G_02205	virB9 protein	ND	-1.27	0.0045	**2.38 (2.42; 2.35**)	**1.56**	**0.0000**	NS	
A1G_02230	virB10 protein	ND	NS		**1.72 (1.69; 1.76**)	**1.55**	**0.0000**	1.81	0.0000
A1G_02235	type IV secretion system ATPase VirB11	ND	-1.35	0.0002	**1.68 (1.70; 1.66**)	**2.00**	**0.0000**	1.49	0.0000
A1G_02240	type IV secretion system component VirD4	ND	-1.19	0.0196	**1.83 (1.72; 1.94**)	**1.64**	**0.0000**	1.38	0.0000
A1G_02555	thioredoxin peroxidase 1	ND	NS		**1.67 (1.59; 1.75**)	**1.84**	**0.0000**	2.06	0.0000
A1G_02680	16S rRNA-processing protein RimM	ND	NS		**-2.06 (-2.18; -1.93**)	**-2.78**	**0.0000**	-2.33	0.0000
A1G_02785	site-specific tyrosine recombinase XerD	**1.53 (1.53; 1.53**)	**1.86**	**0.0000**	**-2.69 (-2.52; -2.87**)	**-4.35**	**0.0000**	-2.27	0.0000
A1G_03790	cell surface antigen	**-1.79 (-1.99; -1.60**)	**-1.96**	**0.0000**	ND	1.40	0.0002	0.72	0.0000
A1G_05315	chaperonin GroEL	ND	1.46	0.0006	**-1.71 (-1.53; -1.88**)	**-2.27**	**0.0000**	-1.54	0.0000
A1G_05630	cold shock-like protein	**1.92 (1.98; 1.87**)	**1.36**	**0.0063**	ND	-1.96	0.0000	-1.43	0.0002
A1G_05960	bicyclomycin resistance protein	**1.57 (1.60; 1.53**)	**1.82**	**0.0000**	ND	-5.88	0.0000	-3.33	0.0000
A1G_05625	ATP-dependent RNA helicase RhlE	ND	NS		**-2.87 (-3.13; -2.62**)	**-4.76**	**0.0000**	-3.85	0.0000
A1G_06265	ADP,ATP carrier protein	ND	1.47	0.0005	**-2.30 (-2.20; -2.41**)	**-4.17**	**0.0000**	-2.78	0.0000
A1G_06705	NADH dehydrogenase subunit J	ND	1.46	0.0018	**-2.02 (-2.11; -1.93**)	**-4.17**	**0.0000**	-2.86	0.0000
A1G_06710	NADH dehydrogenase subunit K	ND	NS		**-4.08 (-4.59; -3.56**)	**-12.5**	**0.0000**	-10.00	0.0000
A1G_06715	NADH dehydrogenase subunit L	ND	1.46	0.0124	**-1.75 (-1.97; -1.54**)	**-7.14**	**0.0000**	-5.00	0.0000
A1G_07470	DNA mismatch repair protein	**-1.62 (-1.69; -1.54**)	**-1.22**	**0.0481**	ND	1.32	0.0001	NS	
A1G_00825	hypothetical protein A1G_00825	ND	1.62	0.0012	**-1.68 (-1.77; -1.59**)	**-3.03**	**0.0000**	-1.85	0.0000
A1G_02840	hypothetical protein A1G_02840	ND	NS		**-3.03 (-3.30; -2.77**)	**-6.67**	**0.0000**	-7.14	0.0000
A1G_06350	hypothetical protein A1G_06350	ND	NS		**3.57 (4.12; 3.01**)	**1.76**	**0.0000**	1.86	0.0000
A1G_07480	hypothetical protein A1G_07480	**-2.62 (-3.02; -2.21**)	**-1.49**	**0.0001**	**2.51 (2.41; 2.62**)	**2.39**	**0.0000**	1.59	0.0000
A1G_00185	hypothetical protein A1G_00185	ND	NS		**2.02 (2.13; 1.92**)	**2.00**	**0.0000**	1.74	0.0000
A1G_00820	hypothetical protein A1G_00820	ND	1.77	0.0001	**-1.83 (-1.88; -1.78**)	**-4.00**	**0.0000**	-2.22	0.0000
A1G_01380	hypothetical protein A1G_01380	ND	NS		**2.71 (2.69; 2.73**)	**2.35**	**0.0000**	1.96	0.0000
A1G_03000	hypothetical protein A1G_03000	ND	2.53	0.0000	**-7.2 (-8.32; -6.07**)	**-7.14**	**0.0000**	-2.78	0.0000
A1G_06745	hypothetical protein A1G_06745	ND	NS		**2.82 (2.94; 2.69**)	**1.69**	**0.0000**	1.85	0.0000
A1G_05315	chaperonin GroEL	ND	1.46	0.0006	**-1.71 (-1.53; -1.88**)	**-2.27**	**0.0000**	-1.54	0.0000
A1G_05630	cold shock-like protein	**1.92 (1.98; 1.87**)	**1.36**	**0.0063**	ND	-1.96	0.0000	-1.43	0.0002

Microarray fold-changes represent the mean of the fold-changes of two biological replicates (1 and 2). ND: differential expression not detected by microarrays. RT-qPCR fold changes (mean of 20 biological replicates) with statistically significant differences with respect to are presented. NS: not significant differences in relation to control. [p<0.05; multiple comparisons by the False Discovery Rate (FDR) method]. Control: unfed ticks at 25°C (G1) for temperature upshift and combined effects of temperature upshift and blood feeding; unfed ticks at 35°C (G2) for blood feeding. Bold values represent genes with the same transcriptional pattern in both microarray and RT-qPCR experiments.

**Table 2:** Transcript levels of selected genes of *R. rickettsii* analyzed by high-throughput microfluidic RT-qPCR.

		**Temperature shift**		**Blood feeding**		**Temperature shift + Blood feeding**	
**Gene ID**	**Annotation**	**Fold change**	***p*-value**	**Fold change**	***p*-value**	**Fold change**	***p*-value**
A1G_00545	stage 0 sporulation protein J	-1.18	0.0058	-1.16	0.0047	-1.39	0.0000
A1G_00890	preprotein translocase subunit SecF	NS		-1.43	0.0000	-1.41	0.0000
A1G_01005	preprotein translocase subunit SecE	1.44	0.0000	-1.25	0.0003	1.15	0.0047
A1G_01150	outer membrane protein omp1	NS		-1.59	0.0000	-1.49	0.0000
A1G_01255	ankyrin repeat-containing protein	1.52	0.0017	-1.52	0.0013	NS	
A1G_01335	molecular chaperone DnaK	-1.41	0.0003	NS		-1.27	0.0094
A1G_01500	chaperone protein HscA	-1.16	0.0196	-1.37	0.0003	-1.61	0.0000
A1G_01505	co-chaperone HscB	1.70	0.0000	-1.37	0.0001	1.24	0.0088
A1G_02285	cysQ protein	1.34	0.0023	-1.27	0.0027	NS	
A1G_02675	outer membrane assembly protein	-1.12	0.0351	NS		-1.16	0.0179
A1G_02960	ankyrin repeat-containing protein	-1.67	0.0000	-1.33	0.0008	-2.22	0.0000
A1G_04625	hemolysin	1.21	0.0400	-1.52	0.0003	-1.25	0.0343
A1G_04750	cell division protein FtsL	NS		-1.43	0.0003	-1.41	0.0009
A1G_04855	preprotein translocase subunit SecA	-1.41	0.0000	NS		-1.25	0.0039
A1G_04935	preprotein translocase subunit YajC	1.17	0.0145	1.28	0.0001	1.50	0.0000
A1G_04940	preprotein translocase subunit SecD	-1.35	0.0001	NS		-1.27	0.0001
A1G_05085	patatin b1 precursor	NS		-1.59	0.0000	-1.85	0.0000
A1G_06030	outer membrane protein B (cell surface antigen sca5)	NS		1.49	0.0000	1.56	0.0000
A1G_06165	DNA polymerase III subunit epsilon	NS		-1.85	0.0000	-2.04	0.0000
A1G_06915	cell surface antigen-like protein Sca13	NS		-1.32	0.0000	-1.35	0.0000
A1G_07170	type II citrate synthase	-1.2	0.0038	NS		-1.28	0.0004
RrIowa_1080	rickA Arp2/3 complex activation protein	-1.23	0.0227	-2.5	0.0000	-3.03	0.0000
A1G_00180	hypothetical protein A1G_00180	1.18	0.0241	1.61	0.0000	1.89	0.0000
A1G_00745	hypothetical protein A1G_00745	NS		-1.33	0.0449	NS	
A1G_03155	hypothetical protein A1G_03155	NS		-1.75	0.0000	-1.79	0.0000
A1G_04930	hypothetical protein A1G_04930	1.23	0.0068	1.24	0.0022	1.53	0.0000

RT-qPCR fold changes (mean of 20 biological replicates) with statistically significant differences with respect to control are presented. NS: not significant differences in relation to control. Control: unfed ticks at 25°C (G1) for temperature upshift and combined effects of temperature upshift and blood feeding; unfed ticks at 35°C (G2) for blood feeding. [p<0.05; multiple comparisons by the False Discovery Rate (FDR) method].

## Discussion

Global transcriptional studies are a useful tool to investigate the response of rickettsiae to environmental stimuli using *in vitro* vertebrate and invertebrate host cell cultures [9–12,34]. In this study, we used customized oligonucleotide microarrays to evaluate the global transcriptional profile of the obligate intracellular bacterium *Rickettsia rickettsii* infecting its vector, the *Amblyomma aureolatum* tick. To the best of our knowledge, this is the first report on the gene expression of a highly virulent bacterium in the genera *Rickettsia* infecting a natural tick vector. The genome of the Sheila Smith strain of *R. rickettsii* (GenBank CP000848.1) was used as reference to design the oligonucleotide microarrays. A genomic comparison of this virulent strain with the avirulent Iowa strain had shown 143 deletions and 492 single nucleotide polymorphisms (SNPs) between the two genomes [18]. Among the SNPs, 339 were found in predicted open reading frames (ORFs), but only 188 SNPs result in non-synonymous amino acid substitutions [18]. Moreover, only four genes were differentially expressed by hybridization of the RNA of the virulent strains Sheila Smith or R with microarrays containing both the Sheila Smith and Iowa genomes, indicating that the genetic differences between the two strains do not alter gene expression [18]. 

Because of limited rickettsial RNA in infected ticks, we performed whole transcriptome linear amplification prior to RNA labeling and microarray hybridization. As demonstrated previously [34–36], this procedure is critical for obtaining good transcriptional data. Using this methodology, we investigated the influence of two environmental stimuli, a 10°C temperature elevation and the acquisition of a blood-meal by the tick vector, on *R. rickettsii* global gene expression. In total, 265 genes were detected to be modulated by either the temperature upshift or blood feeding. Thirty five of the 265 modulated genes (>13%) were selected for further high-throughput RT-qPCR analysis. A correlation of 100% between RT-qPCR and microarray data was obtained, which increased our confidence in the microarray data. 

The temperature upshift and blood feeding, which have been previously associated with the reactivation of rickettsial virulence in ticks [3–6], specifically altered the expression of distinct gene sets. Remarkably, blood feeding modulated five-fold more genes than the temperature shift. The substantial change in the *R. rickettsii* transcriptional profile elicited by the blood meal coincided with increased bacterial load in fed compared with starving ticks. Therefore, it is plausible to suppose that nutrients in the blood meal may stimulate bacterial proliferation in fed ticks. In fact, 16 of the 80 genes up-regulated by the blood meal were related to (i) energy production and conversion or (ii) replication, recombination, and repair. Nonetheless, genes involved in translation, ribosomal structure, and biogenesis are mostly down-regulated. Therefore, we can hypothesize that *R. rickettsii* may repress translation and ribosome structure/biogenesis genes when nutrients are abundant to avoid a disordered proliferation, what could lead to vector’s death. The repression of the 30S ribosomal protein S2 (A1G_00680) was validated by RT-qPCR. We also observed that the encoding genes for two ATP/ADP carrier proteins (A1G_00515 and A1G_06265) and one glycerol-3-phosphate transporter (A1G_00520) are repressed in fed ticks, suggesting that a fewer number of membrane transporter molecules are necessary in a nutrient-rich environment.

**Figure 3:**
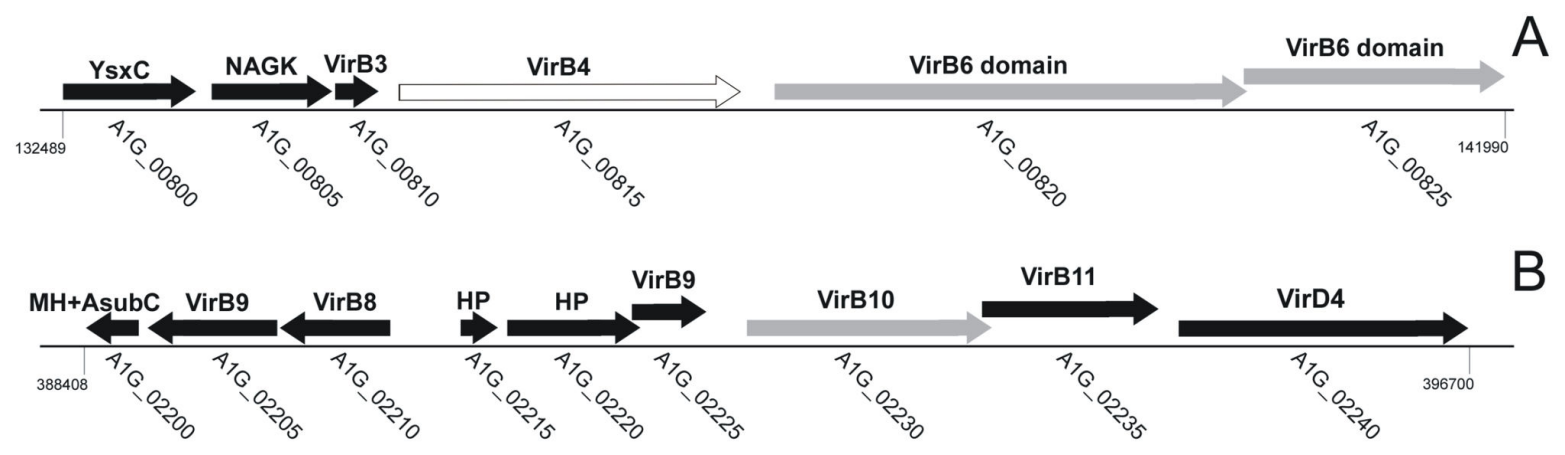
Location and orientation of genes encoding T4SS components repressed (A) or induced (B) by blood feeding in the *R. rickettsii* genome. Black arrows represent genes modulated in two biological replicates and grey arrows genes modulated in only one biological replicate in microarray experiments. The modulation of the ATPase VirB4 (A1G_00815; white arrow) was not observed.

Blood acquisition also up-regulated genes associated with trafficking and secretion, including components of the Type IV Secretion System (T4SS). The T4SS is a protein complex that delivers macromolecules (effectors) from the bacterium to the host cell where they interact with host proteins, enabling bacterial replication and survival [37]. Its importance has been studied in members of the Order Rickettsiales, such as *Anaplasma phagocytophilum* and *Ehrlichia chaffeensis* (for review, see [23]), and in *Coxiella burnetii* [38]. Here, we demonstrated that blood feeding modulated the expression of *R. rickettsii* genes that encode T4SS components. The expression of *virB*3, *virB9*, *virB10*, *virB11*, and *virD4* was statistically validated by microfluidic RT-qPCR. These data indicated that this bacterial transport system might play an important role in secreting rickettsial effectors during blood meal acquisition by the tick vector. However, whereas *virB9*, *virB10*, *virB11*, and *virD4* were induced by this stimulus, *virB3* was repressed. Because *virB3* is located on a different region of the *R. rickettsii* chromosome (Figure 3), it is possible that this gene is controlled by a different regulatory mechanism. Interestingly, two hypothetical protein-encoding genes, A1G_00820 and A1G_00825, that are located downstream of v*irB3* (Figure 3) were also repressed by tick blood feeding, while the modulation of the ATPase VirB4 (A1G_00815) was not detected. These two hypothetical proteins contain VirB6 domains (Tables 2 and S5) and were also statistically validated by RT-qPCR. The presence of two copies of VirB6 in *R. rickettsii* agrees with a previous analysis of T4SS components in the genomes of several species of the genera *Rickettsia* [39]. Additionally, the hypothetical protein A1G_01380 was up-regulated by blood feeding and contains a VirB2 domain. This T4SS component has been previously described to be absent in *Rickettsia* spp. [39].

In contrast to what we observed, the gene expression of T4SS components was reported to be up-regulated during nutrient depletion in *R. conorii*, the causative agent of Mediterranean spotted fever [34]. Nevertheless, in addition to being a different species, the previously published study was performed *in vitro* using a culture of Vero cells as the host. The effects of nutrient deprivation were also investigated in *R. rickettsii* infecting Vero cells [12]. Under limiting iron conditions, five hypothetical protein-encoding genes were modulated with a fold-change ≥ 3.0. One of the genes up-regulated by *in vitro* iron limitation encodes the hypothetical protein A1G_04305 [12]. We demonstrated that this protein encoding gene was repressed when *A. aureolatum* acquired a blood meal, which is a rich iron source. The domain analysis indicated that the hypothetical protein A1G_04305 contains ankyrin repeat domains (Table S5). Ankyrin repeat domains are protein motifs involved in protein-protein interactions that mediate diverse cellular processes [40]. Despite typically being found in eukaryotes, proteins containing ankyrin repeat domains (referred to as Anks) have also been described in bacteria, especially intracellular ones, where they act as T4SS effectors (for review, see [41,42]). Blood feeding down-regulated another gene encoding a hypothetical protein (A1G_02840) with ankyrin repeat domain. It is possible that these two proteins act as T4SS effectors in mammals but not in ticks. The encoding genes of two additional ankyrin-repeat containing proteins (A1G_02960 and A1G_01255) were analyzed by RT-qPCR. Data showed that both genes are repressed by the blood meal, while A1G_01255 is induced by temperature upshift. Therefore, their role as T4SS effectors in host cells should be investigated.

The global transcriptional profile of the etiological agent of Lyme disease, *Borrelia burgdorferi*, has been reported to be modulated by the addition of blood to *in vitro* cultures [43]. The genes encoding the outer surface protein C (OspC) and a Lon protease, an enzyme possibly involved in remodeling the spirochete outer surface, were up-regulated. In *R. rickettsii*, the acquisition of a blood meal by the tick vector stastistically repressed three genes encoding the cell surface protein Sca8, one encoding Sca10, and one encoding Sca13. The Sca (surface cell antigen) group includes the two major *Rickettsia* surface proteins rOmpA (Sca0) and rOmpB (Sca5) (for review, see [44]). rOmpA and rOmpB were previously reported to be involved in the attachment and/or invasion of mammalian cells by rickettsiae [26,29]. In addition, it was demonstrated that the invasion of Vero cells by *R. conorii* involved the interaction of rOmpB with Ku-70, a 70 kDa host protein [30,45]. Although the modulation of rOmpA and rOmpB was not detected by microarray experiments, RT-qPCR analysis showed that rOmpB is up-regulated upon blood feeding alone or combined to temperature upshift. Therefore, studies on the role of rOmpB in invasion of vector and host cells are warranted.

The heme component of the blood meal contributes to the formation of free radicals in blood feeding arthropods (for review, see [46]). Therefore, it is plausible to suppose that *R. rickettsii* may be under oxidative stress in fed ticks. The production of reactive oxygen and nitrogen species (ROS and RNS, respectively) is an ancient immune response to infection and therefore is widespread in the animal kingdom (for review, see [47]). Indeed, the hemocytes of the cattle tick *Rhipicephalus microplus* have been reported to produce ROS when stimulated by bacteria [48]. ROS can oxidize DNA, lipids, and proteins, interfering with their function [49]. Importantly, it has been suggested that bacterial DNA may be the primary target of ROS when free iron concentrations are high [50]. In the current study, we demonstrated that blood feeding statistically induced the expression of three antioxidant enzymes (thioredoxin peroxidase 1, glutaredoxin 3, and ferredoxin) and one hypothetical protein with thioredoxin domain (A1G_00185). It is possible that the induction of these enzymes reflects an attempt by *R. rickettsii* to protect itself against the deleterious effects of free radicals in fed ticks. Interestingly, the glutaredoxin (Grx) and thioredoxin (Trx) systems are involved in returning the cellular proteome to normal after or during unfavorable conditions (for review, see [49]).

The exposure of *R. rickettsii* to a 10°C temperature increase also altered the global transcriptional profile of unfed ticks. This temperature upshift naturally occurs when a starving tick on the ground climbs onto a vertebrate host and contacts its warm skin. This stimulus, compared with blood feeding, did not extensively alter gene transcription. This limited response may partly be explained by the absence of an ideal microenvironment for the bacteria within the starving tick, where energy and nutrients are scarce. A limited gene expression response to temperature shift in *R. rickettsii* infecting a tick cell line (22°C to 37°C) or Vero cells (25°C to 34°C) *in vitro* has been reported [12]. Major gene expression alterations were only detected with larger temperature shifts [12]. In contrast, a temperature downshift (37°C to 25°C) was previously reported to modulate the expression of several genes in *R. typhi* in an *in vitro* cell culture model, down-regulating several chaperonins and heat shock proteins [11]. Under a similar temperature shift (25°C to 35°C), we observed the induction of only one heat shock protein (GrpE) by microarray experiments. The analysis of additional chaperones and heat shock proteins by microfluidic RT-qPCR showed that the encoding genes of the co-chaperone HscB and the chaperonin GroEL are also up-regulated by the temperature elevation, while the molecular chaperone DnaK and chaperone protein HscA are repressed. This indicates that a 10°C temperature elevation does not trigger a heat-shock response in *R. rickettsii* as observed in *R. typhi*. Interestingly, the cold shock-like protein A1G_05630 was up-regulated by a temperature upshift. Because A1G_05630 is the unique cold-shock protein in *R. rickettsii* genome, this role should be addressed.

In summary, we identified distinct effects of blood feeding and a temperature shift on the transcriptional profile of the obligate intracellular virulent pathogen *R. rickettsii* infecting its natural tick vector *A. aureolatum*. Both conditions modulated bacterial gene expression, although the analyses revealed that blood feeding caused more significant effects, for instance, increasing bacterial load. Interestingly, a similar transcriptional profile was observed upon blood feeding alone or combined to the temperature upshift. This result strengthens that the components of blood meal triggers major alterations in rickettsial gene expression modulation than the temperature upshift. These two environmental stimuli simulate the challenges that the bacterium is actually exposed to when infecting its natural tick vectors and have been previously associated with the reactivation of bacterial virulence. Therefore, this is an important approach for identifying key molecules involved in bacterial biological processes and vector interactions that may have potential as targets for vaccine development. The modulated genes identified in this study, including those encoding hypothetical proteins, should be functionally characterized.

## Supporting Information

Table S1 **Primers used in microfluidic RT-qPCR analysis.**
(XLSX)

Table S2 **Genes of *R. rickettsii* encoding proteins with annotated function differentially expressed by the elevation of temperature.**
(XLSX)

Table S3 **Hypothetical protein encoding genes modulated by temperature shift.**
(XLSX)

Table S4 **Genes encoding proteins with annotated function modulated by blood feeding.**
(XLSX)

Table S5 **Hypothetical protein encoding genes differentially expressed by blood feeding.**
(XLSX)
